# Dual RNA-seq transcriptome analysis of caecal tissue during primary *Eimeria tenella* infection in chickens

**DOI:** 10.1186/s12864-021-07959-7

**Published:** 2021-09-14

**Authors:** Arnar K. S. Sandholt, Eva Wattrang, Tobias Lilja, Harri Ahola, Anna Lundén, Karin Troell, Staffan G. Svärd, Robert Söderlund

**Affiliations:** 1 Department of Microbiology, National Veterinary Institute, Uppsala, Sweden; 2 Department of Cell and Molecular Biology, Uppsala University, Uppsala, Sweden

**Keywords:** *Eimeria tenella*, Chicken, Dual RNA-seq analysis, Immune responses

## Abstract

**Background:**

Coccidiosis is an infectious disease with large negative impact on the poultry industry worldwide. It is an enteric infection caused by unicellular Apicomplexan parasites of the genus *Eimeria*. The present study aimed to gain more knowledge about interactions between parasites and the host immune system during the early asexual replication phase of *E. tenella* in chicken caeca. For this purpose, chickens were experimentally infected with *E. tenella* oocysts, sacrificed on days 1–4 and 10 after infection and mRNA from caecal tissues was extracted and sequenced.

**Results:**

Dual RNA-seq analysis revealed time-dependent changes in both host and parasite gene expression during the course of the infection. Chicken immune activation was detected from day 3 and onwards with the highest number of differentially expressed immune genes recorded on day 10. Among early (days 3–4) responses up-regulation of genes for matrix metalloproteinases, several chemokines, interferon (IFN)-γ along with IFN-stimulated genes *GBP, IRF1* and *RSAD2* were noted. Increased expression of genes with immune suppressive/regulatory effects, e.g. *IL10, SOCS1*, *SOCS3*, was also observed among early responses. For *E. tenella* a general up-regulation of genes involved in protein expression and energy metabolism as well as a general down-regulation genes for DNA and RNA processing were observed during the infection. Specific *E. tenella* genes with altered expression during the experiment include those for proteins in rhoptry and microneme organelles.

**Conclusions:**

The present study provides novel information on both the transcriptional activity of *E. tenella* during schizogony in ceacal tissue and of the local host responses to parasite invasion during this phase of infection. Results indicate a role for IFN-γ and IFN-stimulated genes in the innate defence against *Eimeria* replication.

**Supplementary Information:**

The online version contains supplementary material available at 10.1186/s12864-021-07959-7.

## Background

Coccidiosis is regarded as one of the most important infectious diseases in modern poultry rearing with significant impact on animal health, animal welfare and industry economy [1–3]. The infection causes gastrointestinal disease with symptoms ranging from decreased feed conversion to acute deaths. A recent calculation of the costs of coccidiosis prophylaxis, treatment and losses in chickens estimated a yearly global cost of £10.4 billion at 2016 prices [4]. Prophylactic coccidiostat medication and live vaccines are available, but carry drawbacks such as resistance development, limited supply of the vaccines, costs and ethical concerns [3, 5]. Hence, the development of new sustainable control methods is demanded by the industry, but will require a better understanding of the biology of *Eimeria* infection in the chicken host.

The disease is caused by Apicomplexan parasites of the genus *Eimeria* with seven chicken specific *Eimeria* species [1–3]. The clinical outcome of infection varies between the *Eimeria* species and with infection dose, age and immune status of the bird. The life cycle of *Eimeria* is monoxenous and involves three phases: sporulation of oocysts that occur outside the host, asexual replication involving several repeated generations (schizogony), and sexual replication (gametogony), that both occur inside the host. Detailed knowledge on specificities of the *Eimeria* life cycle such as virulence factors, immune evasion mechanisms and proteins recognised by the host immune system is still limited and often inferred from more extensively studied Apicomplexans such as *Toxoplasma gondii*. For example, functions of parasite organelles and proteins from these such as the rhoptry, dense granules and micronemes have been identified with roles in the entry of Apicomplexans into host cells and establishment of the intracellular parasitophorous vacuole [6–9]. Corresponding genes and proteins have in some cases also been identified for *Eimeria*, e.g. rhoptry kinases (ROPK) [10, 11] and micronemes [12, 13]. Moreover, particularly for *T. gondii* several proteins involved in immune recognition and parasite immune evasion have been identified [14, 15] while for *Eimeria* such knowledge is much more limited. Nonetheless, for glycosylphosphatidylinositol (GPI)-anchored surface antigens (SAGs), that have been pointed out in parasite immune recognition, 23 genes have been fully sequenced and identified for *Eimeria tenella* [16]. One of these, *SAG19* has also been structurally defined [17]. Among these *E. tenella* SAGs, SAG4, SAG5, and SAG12 have been shown to be highly immunogenic, stimulate production of nitric oxide, induce expression of *IL1β* and *IL10* and reduce expression of *IL12* and *IFNG* [18].

It is well established that *Eimeria* infection induces species-specific immunity in chickens after single or repeated infections [1, 5]. Protective immunity is thought to be strongly dependent on a Th1-type T-cell response with interferon (IFN)-γ as a key component [5, 19]. Nonetheless, the mechanisms involved in induction of protective immune responses as well as the effector mechanisms involved in parasite control in immune chickens remain largely unknown. The initiation of immune responses is a complex process dependent on both host and parasite traits. Transcriptome analysis may offer a possibility to achieve a more complete picture of complex immune responses, especially in animal species such as the chicken for which the availability of immunological reagents is limited. Earlier studies using microarray-based methodology to study RNA expression in intra-epithelial lymphocytes [20–22] or caecal epithelial cells [23] from *Eimeria* infected chickens have indeed contributed e.g. evidence of the immune pathways activated by *Eimeria* infection such as interleukin and TLR signalling. These studies were however limited to the genes included on the arrays and did not include parasite gene expression. The more recent methodology of RNA-seq offers a comprehensive transcriptome analysis and with dual RNA-seq both host and parasite RNA-expression can be analysed simultaneously, which gives a further dimension to host-parasite interactions [24–28]. We have previously applied this methodology to follow the kinetics of gene expression in chicken macrophage cells infected with *E. tenella* sporozites in vitro during the first round of asexual replication [29]. Results from this study showed early up-regulation of chicken immune related genes at 2–12 h post infection (hpi) followed by strong down-regulation at 24 hpi and a subsequent up-regulation at 72 hpi which coincides with the release of merozoites from first generation schizonts. Results also suggested involvement of pattern recognition receptors (PRRs) mannose receptor C type 2 (MRC2), Toll-like receptor 15 (TLR15) and NOD-like receptor family CARD domain containing 5 (NLRC5) in *E. tenella* innate recognition and revealed *E. tenella* genes such as rhoptry kinases and microneme proteins with distinct expression patterns during this phase of the life cycle. In order to monitor more of the early *E. tenella* life cycle and to include the full host immune system the aim of the present study was to apply the dual RNA-seq methodology in vivo.

*E. tenella* is considered a highly pathogenic species and performs the entire schizogony and gametogony of the life cycle in the caeca of chickens [1–3]. The focus of the present study was immune events and parasite activities during invasion and first and second rounds of asexual *E. tenella* replication, 1–4 days post infection (dpi). Approximately 4 h after ingestion of oocysts *E. tenella* sporozoites invade epithelial cells at the tip of the caecal fold and then migrate to the crypt epithelium where first generation schizonts develop [30]. The mature schizonts rupture and first generation merozoites are released into the caecal lumen and invade epithelial cells. The infected enterocytes subsequently migrate through the basement membrane and second generation schizonts develop in the lamina propria. A third round of asexual replication then takes place in the caecum epithelium before sexual replication commences. For the *E. tenella* Houghton strain used in the present study first generation schizonts are reported first to appear 48 hpi with maximum numbers observed at 60 hpi [31]. The corresponding figures for second and third generation schizonts, respectively, were 84 and 108 hpi for first appearance and 96 and 114 hpi for maximum numbers.

## Results

### Clinical outcome of the *E. tenella* infection

**Fig. 1:**
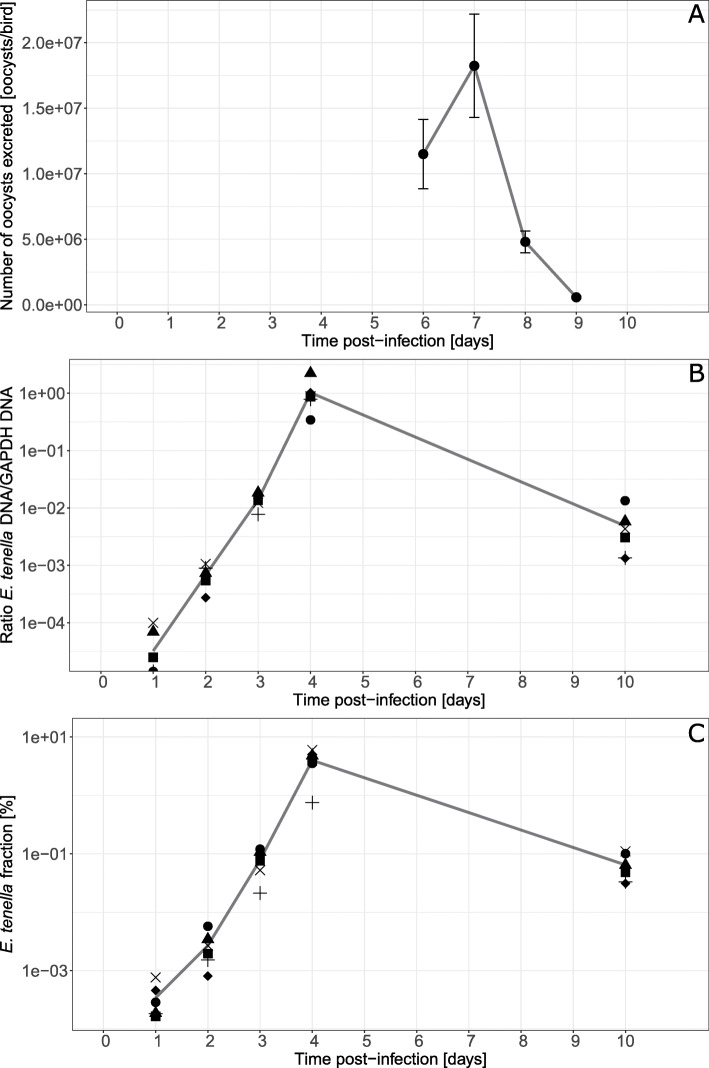
Parasite parameters after experimental infection of chickens with *E. tenella* with 1000 oocysts/bird at day 0. **A** Kinetics of oocyst excretion from 5 to 9 dpi (120–218 h). Results show the mean number of oocysts excreted per bird (±95% confidence intervals, technical replicates *n* = 6 at 6 and 7 dpi and *n* = 3 at 8 and 9 dpi) from faeces collected for 24 h intervals. **B** The ratio of *E. tenella*/chicken GAPDH DNA in DNA samples from chicken caecal tissues collected at the indicated time points post infection and **C** the proportion of *E. tenella* read counts in mRNA samples. Symbols represent individual sample values and the line represents mean values. Corresponding symbols in panel **A**) and **B**) indicate that the RNA and DNA, respectively, was isolated from the same tissue sample

In this study thirty-six chickens were infected with *E. tenella* on experimental day 0 and six were kept as uninfected controls. For tissue sampling uninfected and groups, *n* = 6, of infected chickens were sacrificed on days 1, 2,3, 4 and 10 dpi. Chickens were monitored for clinical signs of disease daily and for oocyst excretion in faeces between 5 and 9 dpi. For infected chickens, blood was observed in faeces from the afternoon of 5 dpi until 7 dpi. At 6 dpi one of the chickens was lethargic and therefore euthanised. The post mortem examination of this chicken showed a caecal lesion score of 4 according to the scoring system of Johnson and Reid [32] with no further pathological findings. For the remaining chickens no other signs of disease were observed. At 7 dpi five chickens were sacrificed and their caecal lesion scores were 3 for four of them and 2 for the remaining one. The oocyst excretion (Fig. 1A) peaked at 7 dpi as expected, but was approximately 100-fold higher at 6 dpi compared to previous results with this infection model [33–35]. The total oocyst yield for the collection period was 3.8 ± 1.4 × 10^7^ oocysts/chicken (mean ± 95% confidence interval), which is well in line with our previous results. The uninfected chickens did not excrete any oocysts.

Caeca sampled in the experiment were macroscopically examined at collection. For caeca collected from uninfected chickens and from the infected chickens at 1 to 3 dpi tissues showed a normal morphology. At 4 dpi caeca from one of the sampled chickens showed a small amount of petechial bleeding in the caecal mucosa. At 10 dpi all caeca were small and contracted with moderately thickened caecal walls and lacked normal content. Cores of white necrotic material (5 of 6 birds) or cores of white and red necrotic material (1 of 6 birds) were present in the caeca and small (3 of 6 birds) or moderate (1 of 6 birds) amounts of petechial bleeding was observed in the caecal mucosa.

Thus, the outcome of the experimental infection was as expected with respect to clinical signs of disease, macroscopic lesions and oocyst excretion.

### Quantification of *E. tenella* DNA in caecal tissues

The amount of *E. tenella* DNA in caecal tissues samples included in the study was assessed with droplet digital PCR (ddPCR; Fig. 1B). At 1 dpi *E. tenella* DNA could be detected in three of the six infected chickens. From 2 dpi and onwards all infected chickens had detectable amounts of *E. tenella* DNA in caecal tissues. Between 1 and 4 dpi the amount of *E. tenella* DNA in the caecal samples increased progressively, approx. 10-fold between 1 and 2 dpi and 2 and 3 dpi, respectively, and approximately 100-fold between 3 and 4 dpi. At 4 dpi the highest amount of parasite DNA was observed for the chicken that also displayed some petechial bleeding in the caecal mucosa. At 10 dpi the amount of DNA in caecal tissues had decreased approx. 100-fold compared to 4 dpi. On this day the chicken with the highest amount of parasite DNA also displayed the most pronounced pathological findings in the caeca at this sampling with moderate petechial bleedings in the mucosa and cores of white and red necrotic material.

Hence, the results showed a pronounced increase in *E. tenella* DNA between 3 and 4 dpi, which corresponds in time with the beginning and peak of the second generation schizonts as reported for the *E. tenella* Houghton strain [31].

### Sequencing and read counting

Sequencing was performed on 36 mRNA samples from the ceaca of uninfected and *E. tenella* infected chickens. Infected samples were from 1, 2, 3, 4, and 10 dpi and six biological replicates were used for each time point and the uninfected sample. The number of reads generated for each time point varied between 15 million and 890 million, with the deepest sequenced samples being from 3, 4, and 10 dpi to achieve informative coverage of *E. tenella* RNA expression at these time points (Additional file 1).

The fraction of *E. tenella* reads varied during the infection, from near zero at 1 dpi to 0.75–6% at 4 dpi (Fig. 1C). Hence, the kinetics of *E. tenella* RNA content corresponds to the kinetics of parasite DNA content in these samples (Fig. 1B). A large of variation between individuals was also observed for the proportion of *E. tenella* RNA content within each time point (Fig. 1C).

### Multidimensional scaling and differential expression analysis

**Fig. 2:**
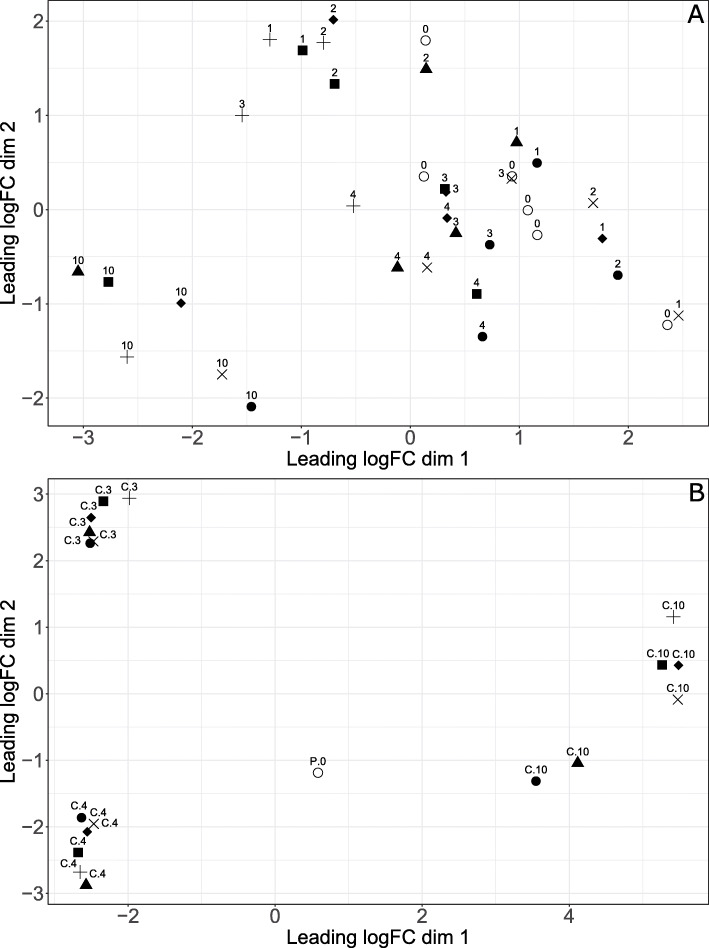
Multidimensional scaling plots for the normalized count data in mRNA samples from caecal tissue. **A** Individual sample values for chicken data in samples collected from uninfected chickens (0) or at the indicated dpi from chickens infected with 1000 *E. tenella* oocysts/bird on day 0. **B** Individual sample values for *E. tenella* data from purified *E tenella* sporozoites (P.0 [29];) or from caecal tissue (**C**) collected at the indicated dpi from chickens infected with 1000 *E. tenella* oocysts/bird at day 0

In order to identify clusters and outliers in the read data, a multidimensional scaling (MDS) analysis was carried out (Fig. 2). Due to the low amount of data on *E. tenella* expression for 1 and 2 dpi, these time points were excluded from the analysis of the parasite data.

For the chicken data, most of the time points did not cluster separately, except for data from 10 dpi (Fig. 2A). The rest of the chicken data formed two somewhat separate clusters, both made up of data from several time points, though one cluster contained all samples from 4 dpi and all but one from 3 and 0 dpi. For *E. tenella* data the opposite was observed with all included time points clustering away from each other (Fig. 2B).

**Fig. 3:**
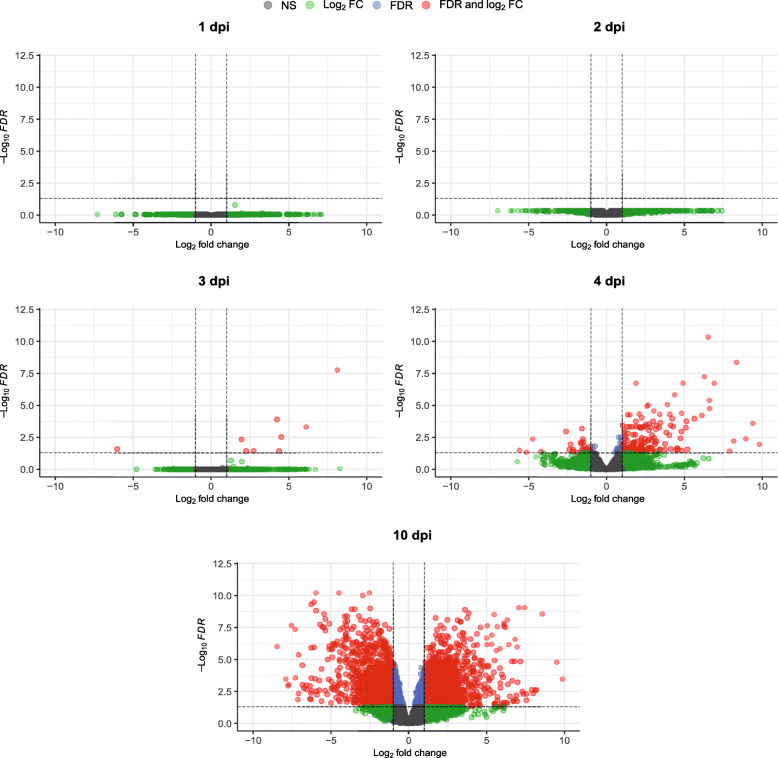
Volcano plots of the differential expression, mRNA from caeca from *E. tenella* infected chickens vs. caeca from uninfected chickens, of all chicken genes at the indicated time points in mRNA samples from chickens infected with 1000 *E. tenella* oocysts/bird at day 0. The significance thresholds were set at log_2_ fold change of ±1 and a false discovery rate of 0.05. NS stands for non-significant

**Fig. 4:**
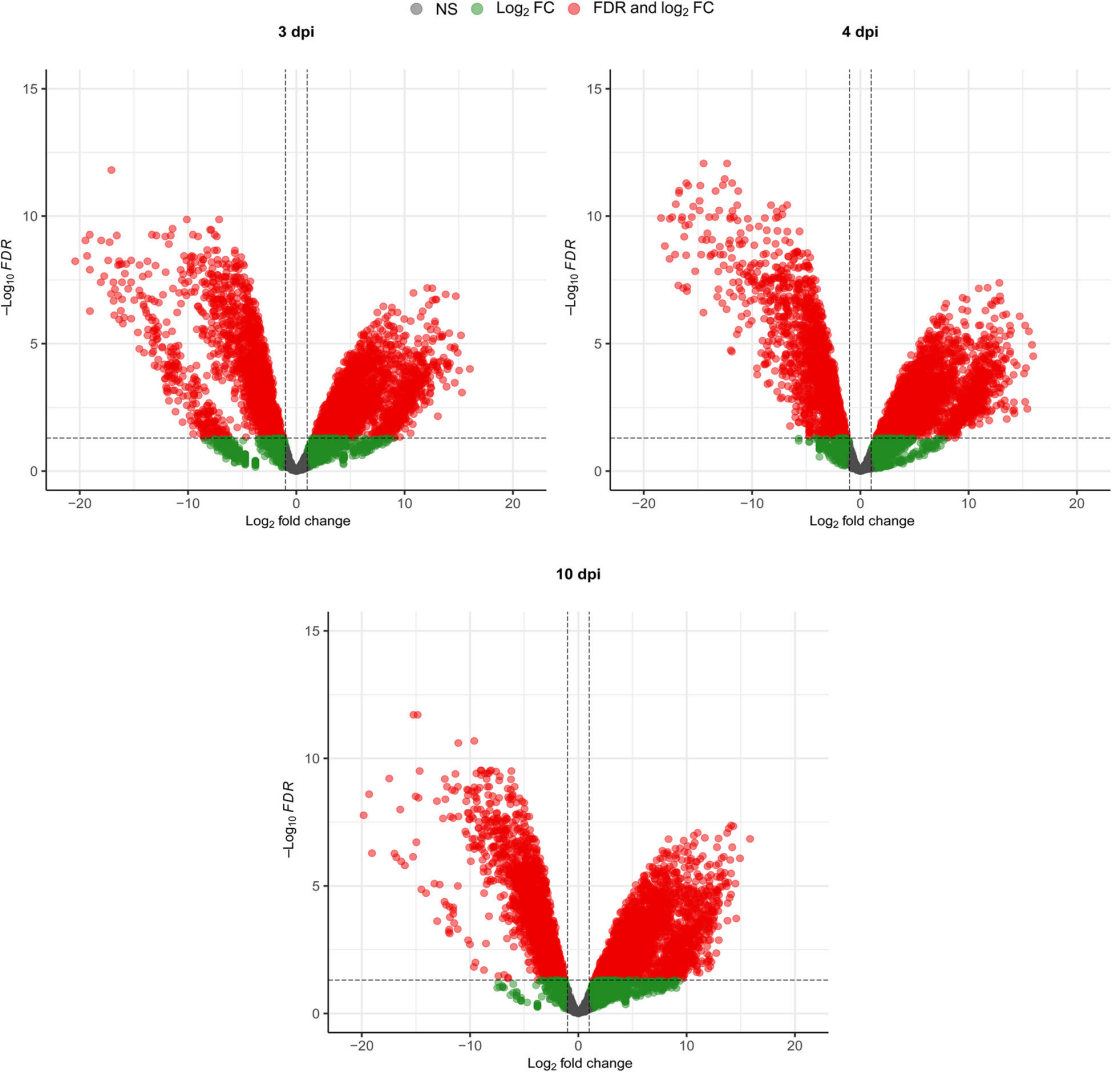
Volcano plots of the differential expression, *E. tenella* mRNA from caeca from *E. tenella* infected chickens vs. *E. tenella* mRNA from sporozoites, of all *E. tenella* genes at the indicated time points in mRNA samples from chickens infected with 1000 *E. tenella* oocysts/bird at day 0. The significance thresholds were set at log_2_ fold change of ±1 and a false discovery rate of 0.05. NS stands for non-significant

Differential expression analysis was carried out for both organisms (Figs. 3 and 4). For the chicken, data from each time point was compared to data from the uninfected chickens. For *E. tenella*, the data was compared to a purified sample of sporozoites, described earlier [29]. For the chicken (Fig. 3) there was no significant differential expression at 1 and 2 dpi and only nine significantly differentially expressed genes at 3 dpi. At 4 dpi, this pattern changed, with several significantly up- and down-regulated genes. Finally, at 10 dpi, a large number of genes were significantly differentially expressed. For *E. tenella*, all included time points show a large number of significantly differentially expressed genes, both up- and down-regulated (Fig. 4).

### Gene ontology (GO) category and Kyoto Encyclopaedia of genes and genomes (KEGG) pathway enrichment analyses

A GO category and KEGG pathway enrichment analysis was carried out for both organisms. The top 50 most significantly enriched categories at each time point can be found in additional files 2, 3, 4 and 5. For the chicken data set GO categories, the top most significantly enriched category was ‘Immune response’ (GO:0006955), being highly up-regulated. Otherwise, the top up-regulated categories at 4 dpi mostly consisted of immune response related processes, such as ‘Interleukin-12 production’ (GO:0032615) and ‘Defense response’ (GO:0006952), while at 10 dpi, cell cycle and repair processes were more dominant, with categories such as ‘Cell cycle process’ (GO:0022402) and ‘DNA recombination’ (GO:0006310).

For chicken KEGG pathways, the most significantly enriched pathways at 4 dpi included ‘Cytokine-cytokine receptor interaction’ (KO:04060), ‘Influenza A’ (KO:05164), and ‘Phagosome’ (KO:04145), all up-regulated. At 10 dpi, ‘Cytokine-cytokine receptor interaction’ remained among the most significantly enriched categories. Only the ‘Metabolic pathways’ (KO:01100) pathway was more significantly enriched, being significantly down-regulated. Other pathways of note include up-regulation of the ‘Toll-like receptor signalling pathway’ (KO:04620), ‘p53 signalling pathway’ (KO:04115), ‘C-type lectin receptor signalling pathway’ (KO:04625) and ‘Intestinal immune network for IgA production’ (KO:04672) as well as down-regulation of the ‘Peroxisome’ (KO:04146) and ‘Ribosome’ (KO:03010) pathways.

For *E. tenella*, the most significantly enriched GO categories at all three time points, 3, 4, and 10 dpi, appeared related to DNA and protein processing and metabolic processes. The most up-regulated categories included ‘Glycolytic process’ (GO:006096), ‘Oxidation-reduction process’ (GO:0055114), ‘Translation’ (GO:0006412), and ‘Protein glycosylation’ (GO:0006486). The most down-regulated categories included ‘Dephosphorylation’ (GO:0016311) and ‘mRNA splicing via spliceosome’ (GO:0000398). The most significantly enriched KEGG pathways showed a similar pattern as the GO categories. ‘Proteasome’ (KO:03050) was significantly up-regulated while ‘Spliceosome’ (KO:03040) was down-regulated across all time points. ‘Ribosome’ (KO:03010) was significantly up-regulated at 3 and 4 dpi but not at 10 dpi. Metabolic pathways such as ‘Glycolysis / Gluconeogenesis’ (KO:00010) and ‘Citrate cycle (TCA cycle)’ (KO:00020) were also up-regulated across all time points. Another pathway of interest, as it is needed for the production of SAGs, is ‘Glycosylphosphatidylinositol (GPI)-anchor biosynthesis’ (KO:00563), which contained up-regulated genes across all time points but the category was only significantly up-regulated at 10 dpi.

### Expression of chicken immune genes and *E. tenella* invasion/infection genes

Separate analyses were undertaken of genes putatively involved in host immune response and parasite infection/invasion processes as described previously [29]. A further manual curation of putative immune response related genes was also carried out.

**Fig. 5:**
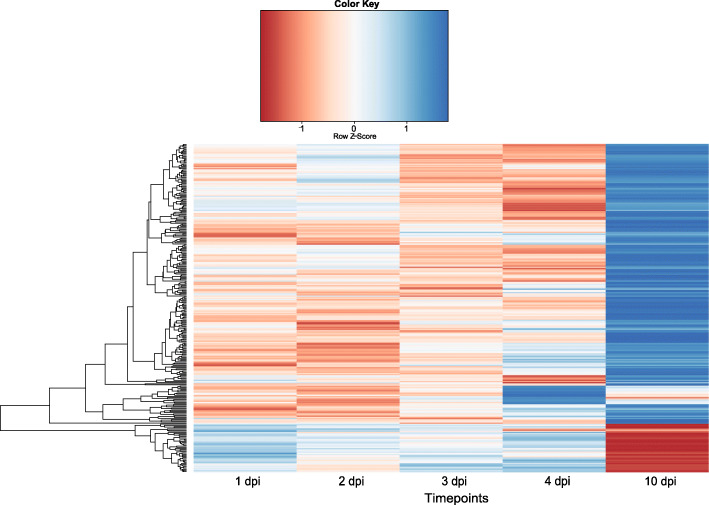
The heatmap depicts the expression profile of 284 immune related chicken genes at the indicated time points in mRNA samples from caecal tissue of chickens infected 1000 *E. tenella* oocysts at day 0. Blue represents up-regulation and red down-regulation. Expression is normalized within each row. For details on the selection of immune related genes see [29]

Results from the differentially expressed chicken immune genes were plotted in a heatmap (Fig. 5). The most pronounced contrasts in the heatmap were the general differences in expression between 1 and 4 dpi and 10 dpi, and more specifically in expression of immune genes between 4 and 10 dpi. Nonetheless, a group of immune genes that were up-regulated at 4 dpi and mostly down-regulated at other time points, including 10 dpi was also observed.

**Fig. 6:**
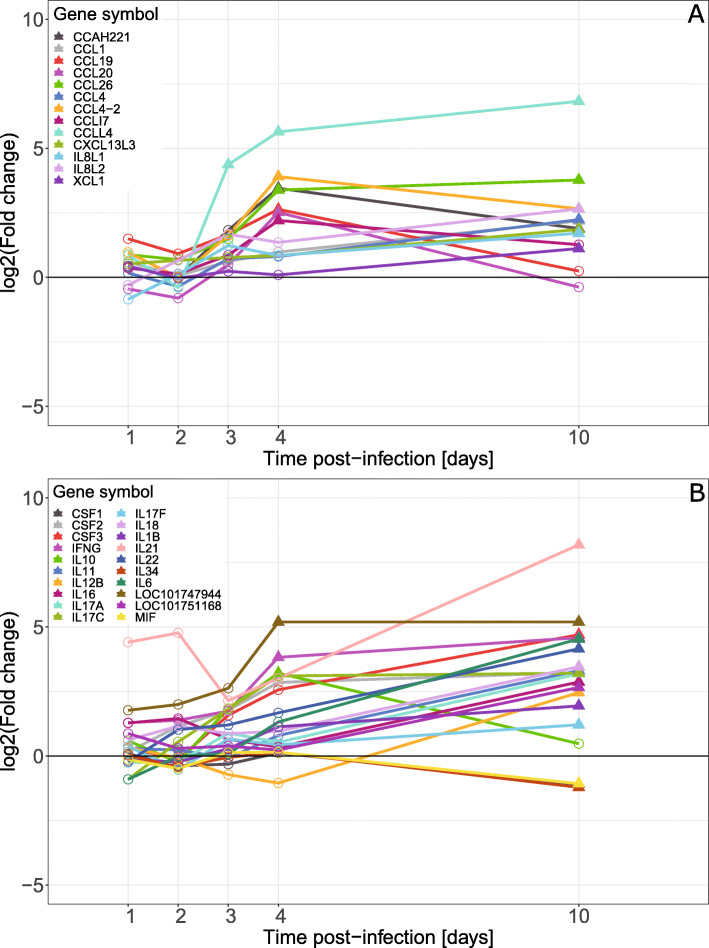
Differential expression, mRNA from caeca of *E. tenella* infected vs. uninfected chickens, of **A** chemokine genes and **B** cytokine genes in samples from chickens infected with 1000 *E. tenella* oocysts/bird at day 0. Point shapes indicate significance, filled triangles for FDR < 0.05 and circles for FDR > 0.05, at the indicated time point

The expression profiles of manually curated categories of host immune genes were plotted only including genes that showed significant differential expression at least at one time point. Among genes for chicken chemokines (Fig. 6A), *CCLL4* showed a distinct expression pattern with significant up-regulation of expression already at 3 dpi and progressively increasing levels of expression at 4 and 10 dpi. For the other chemokines with significant differential expression, two groups with similar intra-group expression patterns were observed, both showed up-regulated expression but at 4 dpi or 10 dpi, respectively. The group up-regulated at 4 dpi included e.g. *CCL4–2* and *CCL19*, and most of these genes showed lower expression at 10 dpi compared to that at 4 dpi. The group with up-regulated expression at 10 dpi, such as *IL8L1* and *IL8L2*, were only significantly up-regulated at this time point.

Among chicken cytokines with significant differential expression the majority were up-regulated and only at 10 dpi (Fig. 6B). However, *IFNG*, *IL10* and *LOC101747944* (IL-12β-like) showed significant up-regulation at 4 dpi and among these solely *IL10* was not significantly up-regulated at 10 dpi. Among cytokines only *MIF* and *IL34* were significantly down-regulated and this occurred at 10 dpi.

**Fig. 7:**
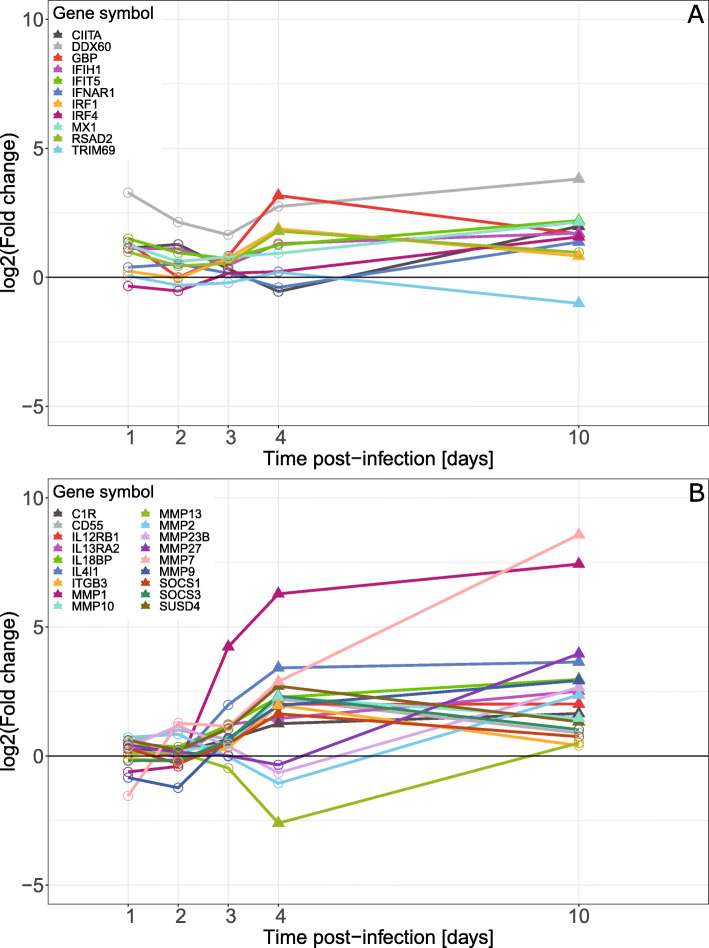
Differential expression, mRNA from caeca of *E. tenella* infected vs. uninfected chickens, of **A** IFN-induced genes and **B** matrix metalloproteinase genes and additional immune related genes significantly differentially expressed at 3 and 4 dpi in samples from chickens infected with 1000 *E. tenella* oocysts/bird at day 0. Point shapes indicate significance, filled triangles for FDR < 0.05 and circles for FDR > 0.05, at the indicated time point

Differentially expressed IFN-stimulated genes were also identified (Fig. 7A) and most of these were significantly up-regulated at 10 dpi only. However, *GBP*, *IRF1* and *RSAD2* were significantly up-regulated at 4 dpi.

For differentially expressed PRR genes (Additional file 6) most were significantly up-regulated at 10 dpi only.

Differentially expressed genes associated with cytotoxic T-lymphocytes (CTL), e.g. the α- and β-chains of the CD8 co-receptor and components of cytotoxic granules, were also identified (Additional file 6). Most of these were significantly up-regulated at 10 dpi only but *CD8A* was significantly up-regulated also at 4 dpi and *LOC100858579* (granzyme G-like) was significantly down-regulated at 10 dpi only.

To broaden the focus on early immune activation we also examined immune related genes with significant differential expression at 3 and 4 dpi that did not fall into any of our other studied immune gene categories (Fig. 7B). This analysis revealed that *MMP1* was significantly up-regulated at 3 dpi and the expression continued to increase at 4 and 10 dpi. Other genes for members of the matrix metalloproteinase family with significant differential expression where either significantly up-regulated at 4 and 10 dpi, *MMP7* and *MMP10*, only up-regulated on 10 dpi, *MMP2*, *MMP9*, *MMP23B* and *MMP27*, or down-regulated on 4 dpi, *MMP13*. Among other immune genes with increased expression at 4 dpi also *ITGB3*, *IL21RB1*, *IL13RA2*, *SOCS1*, *SOCS3*, *SUSD4*, *IL4I1*, *IL18BP* and *CD55*, were identified.

**Fig. 8:**
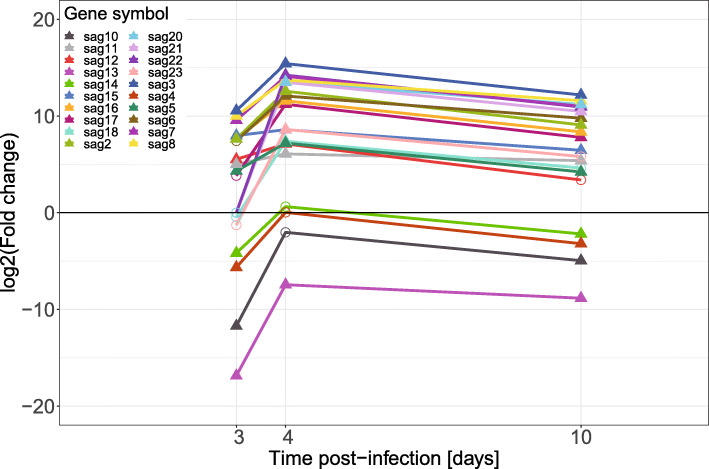
Differential expression of *E. tenella* glycosylphosphatidylinositol-anchored surface antigen (SAG) genes, in mRNA from caeca of *E. tenella* infected chickens vs. *E. tenella* mRNA from sporozoites, in samples from chickens infected with 1000 *E. tenella* oocysts/bird on day 0. Point shapes indicate significance, filled triangles for FDR < 0.05 and circles for FDR > 0.05, at the indicated time point

Several categories of *E. tenella* genes involved in the host cell invasion process of the parasite were also examined: The SAG genes, the rhoptry kinase (ROPK) genes, the rhoptry neck protein (RON) genes, the dense granule (GRA) genes and the microneme (MIC) genes. This analysis made use of the same classification of genes as described previously [29] and parasite gene expression was examined with the same significance thresholds as the chicken genes. The majority of SAG genes (Fig. 8) had similar patterns of expression, with peak expression at 4 dpi and a slight decrease in expression at 10 dpi compared to 4 dpi. Most of the genes were up-regulated with only *SAG14*, *SAG4*, *SAG10* and *SAG13* significantly down-regulated, all at 3 and 10 dpi and *SAG13* at 4 dpi as well.

**Fig. 9:**
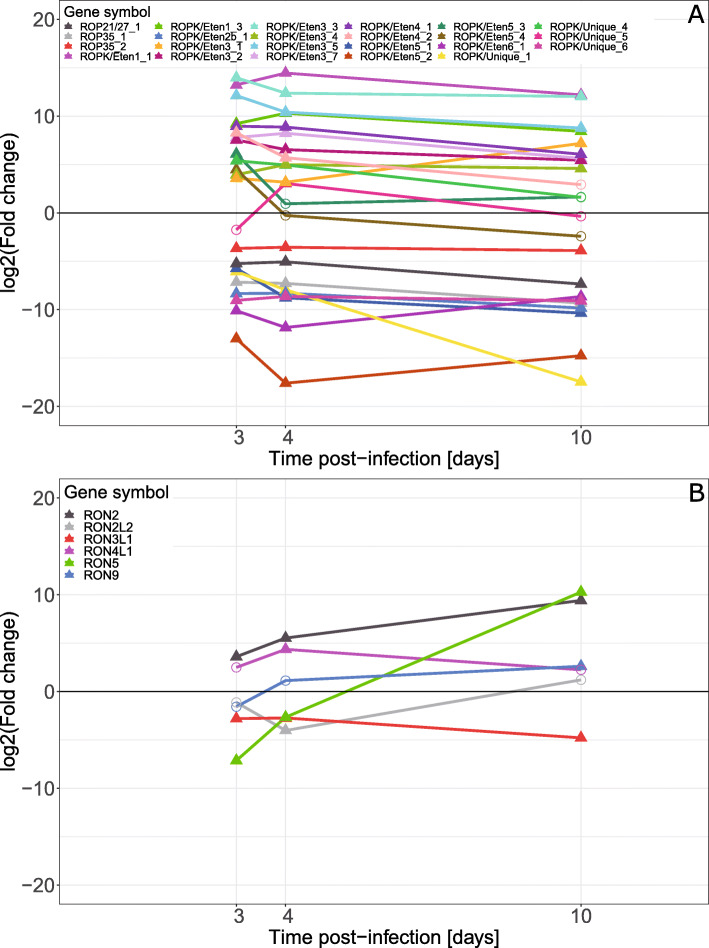
Differential expression of *E. tenella* **A** rhoptry kinase (ROPK) genes, and **B** rhoptry neck protein (RON) genes in mRNA from caeca of *E. tenella* infected chickens vs. *E. tenella* mRNA from sporozoites, in samples from chickens infected with 1000 *E. tenella* oocysts/bird on day 0. Point shapes indicate significance, filled triangles for FDR < 0.05 and circles for FDR > 0.05, at the indicated time point

The ROPK genes (Fig. 9A) tended to be consistently either up-regulated or down-regulated throughout the experiment, compared to that in pre-infection sporozoites. A few, such as *ROPK/Eten3_1* and *ROPK/Unique_1*, showed larger changes, with the first increasing expression at 10 dpi and the latter with decreasing expression during the experiment.

The six differentially expressed RON genes (Fig. 9B) showed varied expression profiles. For example, *RON5* was significantly down-regulated at 3 and 4 dpi but significantly up-regulated at 10 dpi, *RON2* was significantly up-regulated at all time points while *RON9* was significantly down-regulated at all time points.

**Fig. 10:**
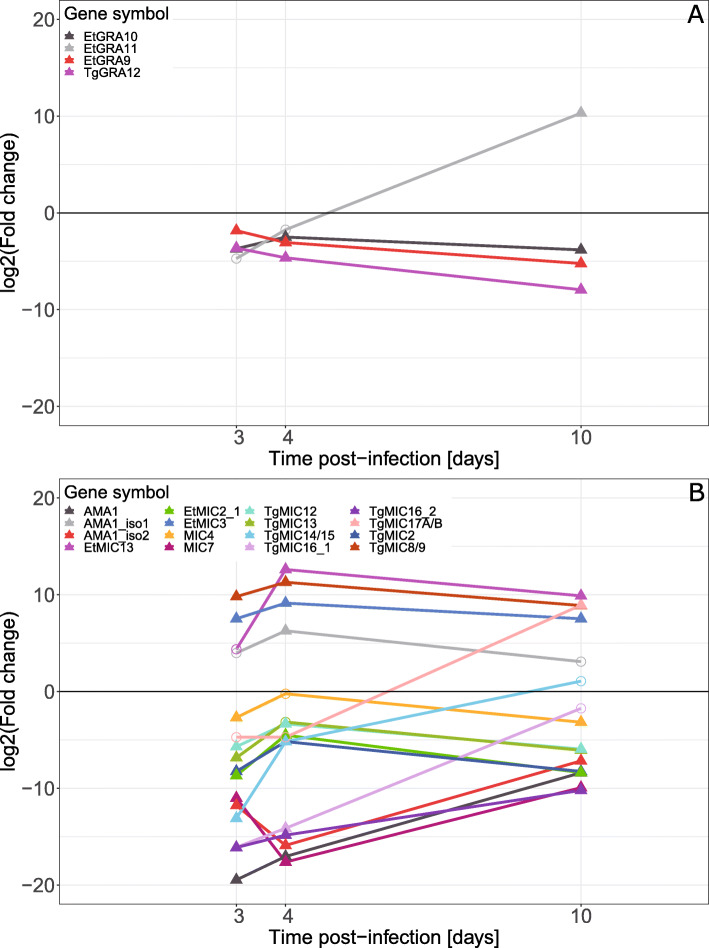
Differential expression of *E. tenella* **A** dense granule (GRA) genes, and **B** microneme (MIC) genes in mRNA from caeca of *E. tenella* infected chickens vs. *E. tenella* mRNA from sporozoites, in samples from chickens infected with 1000 *E. tenella* oocysts/bird at day 0. Point shapes indicate significance, filled triangles for FDR < 0.05 and circles for FDR > 0.05, at the indicated time point

Three of the four differentially expressed GRA genes (Fig. 10A) were down-regulated across all time points while expression of *EtGRA11* progressively increased during the experiment and it was significantly up-regulated at 10 dpi.

The differentially expressed MIC genes (Fig. 10B) had more varied expression patterns. For example *TgMIC8/9* and *EtMIC3* were both significantly up-regulated across all time points while *EtMIC13* was only significantly up-regulated at 4 and 10 dpi, *AMA1_iso1* was significantly up-regulated at 4 dpi and *TgMIC17A/B* at 10 dpi. The rest of the MIC genes were down-regulated across all time points.

## Discussion

This study aimed to contribute to a more comprehensive picture of the transcriptional processes of *E. tenella* and the chicken host immune response during the early phases of infection. We detected significant alterations in the expression of both parasite and chicken genes in the infected caecal tissue from 3 dpi.

For immune responses the focus of the present study was to identify early immune events that reflect the recognition of infection, activation of innate immunity and regulation of ensuing responses. Indeed, regarding overall expression of immune response related genes in caecal tissue a group of genes with up-regulated expression at 4 dpi was identified. Notably, one of the up-regulated GO-categories at this time point was production of interleukin-12, a pro-inflammatory cytokine with key roles in connecting innate immune activation with the initiation of subsequent Th1-type responses [36]. Since Th1-type responses are considered essential for protection against *Eimeria*-infection [19] our results suggest that the chain of events leading to development of protective immunity already was initiated at this time point. This was also supported by a recent report showing that the Th1 pathway was among top up-regulated GO-categories in jejeunal tissue from *E. maxima* infected chickens 4–6 dpi [37]. Beside this, the most striking observation in the general analysis of immune related genes in our data was the up-regulated expression of a large number of genes at 10 dpi. This was an expected observation since at this time the parasite life cycle is completed and many immunological and inflammatory processes are ongoing including tissue regeneration. In this infection model we have previously e.g. observed a prominent increase of CTL among leukocytes in the caecal mucosa [35] and increased mRNA expression of genes for cytotoxic granule proteins such as perforin and granzyme A [33] at 10 dpi. In the current results evidence for the recruitment and activation of CTL was again observed at 10 dpi, as up-regulated expression of e.g. *CD8A*, *CD8B*, *PRF1*, *GZMA*, *GZMK* and *GNLY*, which confirms the sensitivity of the RNA-seq methodology used.

For *E. tenella*, the small amount of genetic material present in the sampled tissue on the first two time points limited the analysis to only 3, 4, and 10 dpi. The data for these three time points, however, was plentiful, with a large number of significantly differentially expressed genes compared to pre-invasion sporozoites. The samples from each of these time points clustered separately in the MDS analysis, showing significant difference in expression patterns. This could be due to difference in parasite expression during the distinct phases of the life cycle. At 3 dpi and 4 dpi, the parasite should be in the midst of the first and second asexual reproduction cycles, respectively [31]. Similarities and distinctions between these two stages might explain why samples from 3 and 4 dpi cluster together along leading logFC dim 1 but form separate clusters along dim 2. At 10 dpi, the life cycle is completed but since oocysts may be detected in faeces up to 14 dpi [31] it is likely that low-grade sexual replication and oocyst maturation still occur. In the present results 10 dpi samples form a looser cluster with “outliers” compared to samples from 3 and 4 dpi. This might indicate that host factors were starting to influence parasite replication leading to a lower degree of synchronisation of replication between individual chickens.

In the overall gene expression analysis a general down-regulation of DNA and RNA processing categories and up-regulation of protein expression and energy metabolism categories was observed. A similar pattern was recorded when the first schizogony of *E. tenella* was monitored in cell culture [29] and analogous results have been observed for *E. maxima* when merozoites and oocysts were compared [38]. Based on these observations one may hypothesise that *Eimeria* merozoites in general may have a lower level of splicing and increased protein expression compared to sporozoites.

In the present study the earliest clear host immune activation observed in caecal tissue was a strong increase in the expression of matrix metalloproteinase *MMP1* and chemokine *CCLL4* at 3 dpi. For *MMP1* a prominent increase in expression was observed from 3 dpi and its expression then remained at a high level throughout the rest of the experiment. In addition, other genes in the matrix metalloproteinase family also showed increased expression at 4 and/or 10 dpi. Matrix metalloproteinases are proteolytic enzymes that have many roles in the immune responses to infections, e.g. by recruiting immune cells, modulating chemokine and cytokine responses and in tissue degradation and remodelling [39]. Earlier reports have shown mRNA expression of several MMPs including MMP1 in caecal epithelial cells [23] and increased levels of serum MMP9 [40] after *E. tenella* infection of chickens and MMP activity has been shown in jejunal content after *E. maxima* infection of chickens [41]. In our previous in vitro study of *E. tenella* infected chicken macrophages [29], *MMP9* expression was transiently up-regulated at 12 hpi while *MMP10* and *MMP17* expression was up-regulated later in the infection at 48–72 hpi (unpublished observation). Hence, considering the prominent early response observed in the present study matrix metalloproteinases may be of importance in the early recognition of *Eimeria* infections. It is also possible that the early, 3 dpi, expression of *MMP1* could be involved in the migration of merozoite infected enterocytes through the basement membrane, which occurs at this time point [30]. In support of this, a transient accumulation of mucosal mast cells has been observed around epithelial cells invaded by first generation *E. tenella* merozoites [30, 42] and it has been suggested that mast cells may disrupt the mucosal basement membrane by activation of matrix metalloproteinases [30, 43].

The chemokine *CCL4* also showed a prominently increased expression at 3 dpi and then remained the chemokine with the highest differential expression throughout the rest of the experiment. At 4 dpi we also observed a group of chemokines, *CCL4–2*, *CCL17*, *CCL19*, *CCL20*, *CCL26* and *CCAH221*, with a clear peak of up-regulated expression. We earlier observed prompt up-regulation of the expression of several chemokines including *CCL4–2*, *CCL17* and *CCL20* at 4 h after in vitro *E. tenella* infection of chicken macrophages [29]. Other reports have also shown increased chemokine gene expression, e.g. IL-8 and CCL4, in intestinal tissues/cells approx. 4 to 10 days after *Eimeria* infection of chickens [23, 44–49], which is in line with the present results. Chemokines are crucial for recruitment of immune cells to the site of infection and important for the regulation of subsequent immune responses. Thus, the present results contribute to a more comprehensive picture of the kinetics of chemokine expression in the initiation of chicken immune responses to *Eimeria* infections.

For cytokines most genes with significantly altered expression were up-regulated as part of the general immune activation at 10 dpi. However, expression of three cytokines were significantly up-regulated at 4 dpi; *IFNG*, *IL10* and *LOC101747944* (IL-12β-like). Among these, IFN-γ is regarded as a cytokine of central importance in chicken immunity to *Eimeria*-infection both as an effector cytokine inhibiting parasite intracellular development and as a key regulator of Th1-type responses (reviewed in [19]. In line with the present results, early IFN-γ responses in intestinal tissues/cells upon primary *Eimeria*-infection of chickens have been reported previously [21, 23, 37, 45, 47, 48, 50, 51]. Moreover, in the present results some of the differentially expressed IFN-induced genes, i.e. *GBP*, *IRF1* and *RSAD2*, were significantly up-regulated at 4 dpi, which may be a result of IFN-γ stimulation. Expression of IFN-induced genes were also reported in caecal epithelial cells 4.5 days after *E. tenella* infection [23] and in jejeunal tissue 4–6 days after *E. maxima* infection [37] and in the latter study IFN signalling was the top enriched GO pathway at 4 dpi for chickens relatively resistant to the infection. For related Apicomplexan parasites *T. gondii* and *Neospora caninum* it has been shown that IFN-γ induced members of the murine guanylate-binding protein (GBP) family can restrict the intracellular growth of the parasites [52], which for *T. gondii* has been suggested essential for survival of infected mice [53]. Also the IFN-induced transcription factor IFN regulatory factor 1 (IRF1) is suggested to be of importance in parasite defence since it has been shown that IRF1 deficient (IRF1^−/−^) mice have a high susceptibility to *T. gondii* infection [54, 55]. In addition, parasite evasion mechanisms aimed at GBP and IRF1 have been described for *T. gondii*. For example, it has been shown that the rhoptry protein TgROP18 inhibits both murine GBPs [14] and human IRF1 [56]. Thus, the early IFN-γ response and IFN-induced innate effector functions may be crucial events in the chicken immune response to *Eimeria*-infection and it would be valuable to identify these early IFN-γ producing cells for our further understanding of *Eimeria* immune recognition.

At 4 dpi we observed a transient up-regulation of *IL10* expression in caecal tissue. Interleukin-10 (IL-10) is a pleiotropic cytokine well known for its immunoregulatory effects and in the context of protozoan infections IL-10 is suggested to have a role in controlling the potentially host-damaging effects of parasite-eliminating immune responses [57, 58]. Induction of IL-10 has earlier been observed upon *Eimeria* infections of chickens [37, 45, 48, 50, 59–61] and increased IL-10 expression has been associated with increased susceptibility to *Eimeria*-infection [37, 50, 59, 61]. Chicken macrophages have also been shown to up-regulate *IL10* expression upon in vitro stimulation with recombinant *E. tenella* surface antigens, SAG4, SAG5 and SAG12, [18]. For these SAGs we observed up-regulated expression of *SAG5* and *SAG12* and down-regulated expression of *SAG4* by the parasite during the present experiment. Hence, the observed IL-10 response may be part of *E. tenella* immune evasive mechanisms as well as part of the host’s intrinsic regulatory immune mechanisms. Interestingly, at 4 dpi several of the other immune related genes with up-regulated expression have general or more targeted immune regulatory effects, i.e. inhibitors of the cytokine-induced STAT cell signalling pathway *SOCS1* and *SOCS3* [62], regulators of specific immune events, e.g. Th1-type responses, *IL4I1* [63] and *IL18BP* [64], and complement inhibitors *SUSD4* [65] and *CD55* [66]. These early events may equally be part of the parasite’s or the host’s defence strategy. As an example of this the expression of suppressor of cytokine signalling (SOCS) 1 [67, 68] is induced by some *T. gondii* strains to avoid the effects of IFN-γ while SOCS3 on the other hand is essential for mounting protective immune responses against *T. gondii* [69], which highlights a complex balance of measures and countermeasures in infection biology.

The expression of several categories of genes involved in the *E. tenella* infection process, i.e. SAG, ROPK, RON, GRA and MIC genes, was examined in greater detail. In the present study, all SAGs, except for *SAG1*, *SAG9* and *SAG19*, showed some level of differential expression. The majority of SAGs showed only up-regulation, indicating roles in the merozoites. *SAG5* and *SAG12* were both up-regulated at all time points while *SAG4* was down-regulated, indicating some expression in the sporozoite. The down-regulation of *SAG10*, *SAG13* and *SAG14* also agrees with previous with the results of Tabarés et al [16] that these are expressed in both the sporozoite and merozoite while the rest were only found in one stage.

Rhoptry proteins ROPK and RON are known to have roles in the initial stages of Apicomplexan cell invasion [7]. For *E. tenella* several sub families of ROPK unique to *Eimeria* have been identified dubbed ROPK/Eten1–6 [11]. In the present study, most of the ROPK genes showed a consistent expression level, with either slightly higher or lower expression at 4 dpi compared to 3 dpi and a similar or slightly lower level at 10 dpi. Overall, the expression of these genes appears to differ far more between the sporozoite and the intracellular lifecycle stages than within the latter. A few genes, such as *ROP35_2*, *ROPK/Eten3_1*, *ROPK/Eten5_2*, *ROPK/Eten5_3*, *ROPK/Eten5_4* and *ROPK/Unique_1* show more dramatic changes in expression level. This may indicate differing roles between the two merozoite stages and between the merozoites and the zygote/developing oocysts at 10 dpi.

The RONs have roles in cell invasion and in formation of the parasitophorous vacuole [7, 10]. In the present study, the RON genes were more varied in expression pattern compared to the ROPK genes, with *RON5* (ETH_00005755), especially, showing a considerable increase in expression between each time point. These results agree with those of Oakes et al. [10], with *RON2* (ETH_00012760) being up-regulated in the merozoites and *RON3L1* (ETH_00007925) being down-regulated (i.e. up-regulated in the sporozoite). *RON5* is interesting as it was highly expressed in the sporozoite, which agrees with it being down-regulated at 3 and 4 dpi. However, it was highly up-regulated at 10 dpi, indicating that it may also play some role during oocyst formation/development.

The dense granule proteins have been extensively studied in *T. gondii* while those of the *Eimeria* genus are still rather poorly defined [6, 9] but several orthologues have been identified in the *E. tenella* genome and were included in the present study. Overall, three of four GRA genes that were significantly differentially expressed were down-regulated at all time points, possibly indicating a larger role in sporozoites than merozoites. The fourth, *GRA11*, was highly up-regulated at 10 dpi, which may indicate a role in the oocyst.

Microneme proteins are involved in cell invasion and formation of the parasitophorous vacuole, e.g. AMA1 [70], as well as parasite motility and binding to host cell membranes [8]. A number of microneme proteins have been described for *E. tenella*, including EtMIC4 [12] and EtMIC2 [13]. In the present study, the three isoforms of *AMA-1* show two different patterns. Both *AMA-1* and *AMA-1_iso2* were significantly down-regulated at all three time points, most strongly at 3 and 4 dpi. *AMA-1_iso1* on the other hand, showed significant up-regulation at 4 dpi. This is in line with previous results, both from our in vitro gene expression experiments [29] and earlier proteomic experiments [71]. It further supports that *E. tenella* uses different forms of AMA1 in sporozoites and merozoites. In addition, an orthologue of *TgMIC17A/B*, showed a similar pattern of expression to *RON5* and *GRA11*, perhaps also indicating a role in the oocyst and sporozoite.

At the two earliest time points in the present study, i.e. 1 and 2 dpi, the proportion of *E. tenella* transcripts was too low to allow meaningful analysis of the expressed genes. Moreover, we did not detect any major changes in the chicken transcriptome at these early time points either. It would be reasonable to expect some host responses to the first generation of schizonts developing during this time. However, with the inoculation dose used a maximum of approx. eight thousand infected enterocytes can be generated and the present methodology might simply not be sensitive enough to detect responses to such a small proportion of the total cells present in the sample. In previous studies using this infection model we did not detect altered expression of CTL associated genes [33] or signs of activation of CD8β-expressing cell populations [35] in caecal mucosa during this phase of infection of naïve chickens, which is in line with the present observations. Among earlier RT-PCR or RNA-seq based studies of *Eimeria*-infected chickens that include samples collected at 1 and 2 dpi, those using intestinal tissue samples reports no or very low changes in mRNA expression at these time points [37, 46, 47] while those using purified intestinal mononuclear leukocytes report genes with altered expression [45, 51]. In addition, RNA-microarray based studies of *Eimeria*-infected chickens using purified intestinal mononuclear leukocytes report some altered gene expression at 1 and 2 dpi [20–22]. However, such studies will of course only allow expression profiling of the purified cell types. It might be more fruitful to study responses upon the first invasion of host cells by sporozoites and during the first round of schizogony in in vitro systems such as the chicken macrophage cell-line we have used earlier [29].

## Conclusions

Taken together this study has given novel insights into the initial immune activation and parasite activities during primary *E. tenella* infection. For example findings suggest a role for IFN-γ and IFN-induced genes as anti-parasite effector mechanisms in the innate chicken defence against *Eimeria* infections. Our results demonstrate the usefulness of dual RNA-seq for addressing the complexity of host-parasite interaction. Future applications of this methodology may include studies of additional *E. tenella* life cycle stages in the chicken host, of other *Eimeria* species and of infections in immune birds.

## Materials and methods

### Maintenance of the *E. tenella* isolate and generation of sporulated oocysts

A pure *E. tenella* Houghton strain [31] isolate was maintained by twice yearly passage in chickens and sporulated oocysts were prepared according to earlier described protocols [33].

### Experimental design and infection of chickens with *E. tenella* oocysts

For this study in total 42 female Dekalb White Leghorn-type laying chickens purchased from a commercial hatchery were used. All chickens were reared from day-old under SPF-conditions at the National Veterinary Institute animal facilities and were group housed in cages in rooms under negative pressure ventilation. At 12 days of age blood samples were collected and analysed for maternal antibodies to *E. tenella* using an in house ELISA as previously described [33]. Chickens were then allocated to seven groups with respect to an even distribution of maternal antibodies. One group was kept as uninfected control and at 17 days of age chickens in the remaining six groups were inoculated orally with 1000 live *E. tenella* oocysts per bird. Five infected groups and the uninfected group were used for tissue sampling and the remaining infected group was used for caecal lesion scoring [32] at 7 dpi.

Viability of inoculation oocysts was estimated based on our experience that approx. 12% of oocysts loose infectivity per month of storage in 2% dichromate at 4 °C and oocysts used in this experiment had been stored for 3 months.

From 5 to 9 dpi all faeces produced by chickens were collected once a day and numbers of oocysts per gram faeces (OPG) were determined as described earlier [33, 34].

### Caecal sample collection and RNA extraction

At 1, 2, 3, 4 and 10 dpi all chickens in one of the infected groups and one or two of the uninfected control chickens were killed by cervical dislocation and the two caeca were collected. Approximately 1 cm of the proximal part of the caeca containing the caecal tonsils was cut off. The remaining caeca were cut open longitudinally and caecal contents were thoroughly rinsed off with ice cold PBS. Ceacal tissues were cut into 0.5 cm wide strips and the tissue from the two caeca from each bird were pooled in 5 ml RNAlater (ThermoFisher Scientific). Samples were subsequently stored at 4 °C for 24 h and thereafter stored at − 20 °C.

Caecal tissues stored in RNAlater were thawed, removed from the solution and briefly air-dried. Tissues were shaken three times in a 15 ml screw cap tube for 60 s at 6.2 m/s in 10 ml of TRIzol (ThermoFisher Scientific) and a 1:1 mixture of 2 mm ø and 0.5 mm ø Zirconia/Silica beads (BioSpec products). After this treatment all mucosal tissue, but not all connective tissue, was homogenized. Samples were then stored at − 70 °C. Total RNA was isolated with a TRIzol/choloroform extraction protocol, DNAse treated, quality controlled and quantified as previously described [29].

### RNA sequencing

RNA samples isolated from the caecum from the uninfected chickens and *E. tenella* infected chickens at 1, 2, 3, 4 and 10 dpi, respectively, were sequenced with six biological replicates for each time point as described above. The sequencing libraries were prepared from 0.4–1.0 μg total RNA using the TruSeq stranded mRNA library preparation kit (Illumina) with polyA selection according to the manufacturer’s protocol. For infected chicken samples collected at 3 and 10 dpi, due to the quantity of data to be generated, 2 sequencing libraries were prepared for each sample to ensure sufficient diversity. Sequencing was done on a NovaSeq 6000 using an S4 flow cell, except for uninfected samples and those from 1 and 2 dpi for which an SP flow cell was used, with paired-end 150 bp reads and v1 sequencing chemistry.

### Read counting and differential expression analysis

Read counting and differential expression analysis was performed as previously described in [29]. Briefly, the reads were quality checked using FastQC v0.11.8 [72] and MultiQC v1.8 [73], trimmed with Trimmomatic v0.36 [74], mapped to the reference genomes (*Gallus gallus*; GCF_000002315.6_GRCg6a, and *Eimeria tenella*; GCF_499545.2_ETH001) using STAR v2.7.2b [75] and mapped reads counted using HTSeq v0.9.1 [76], edgeR v3.28.1 was used to perform differential expression analysis, analysing *E. tenella* and chicken expression separately. The comparisons made were infected tissue samples at each time point vs the uninfected tissue for chicken data and parasites at the infection time points vs a pure sporozoite sample for *E. tenella* data. GO and KEGG enrichment analysis and visualisation of results were performed as previously described [29].

### DNA isolation and ddPCR assays for quantification of *E. tenella* and chicken cells

DNA was isolated from 1 ml aliquots of caecal tissue homogenised in TRIzol according to the TRIzol manufacturer’s protocol. DNA preparations were subsequently cleaved using Bam HI restriction endonuclease (New England BioLabs Inc.) according to the manufacturer’s instructions.

Two ddPCR assays were set up to quantify host and parasite cells using previously described primers and probes for detection of *E. tenella* [77] and chicken glyceraldehyde 3-phosphate dehydrogenase (GAPDH) [33]. The assays were set up for the QX100 ddPCR system according to the Droplet Digital PCR Applications Guide (BIO-RAD). In brief, reactions had a total volume of 20 μl containing 10 μl ddPCR Supermix for Probes (BIO-RAD) 0.4 μM of each primer, 0.13 μM of probe and 2 μl of DNA samples. PCR reactions were carried out in a thermal cycler at PCR cycling parameters 10 min at 95 °C followed by 50 cycles of 30 s at 94 °C and 120 s at 58 °C followed by 10 min at 98 °C. Data were analysed using QuantaSoft software version 1.5.38.1118. FAM fluorescent droplets were analysed in channel 1 and fluorescent and non-fluorescent droplets were separated with a threshold set at 2000 AU. Samples with less than 3 positive droplets were considered negative. Data was expressed as the ratio of copies of *E. tenella*/copies of chicken GAPDH in each sample.

## Supplementary Information

**Additional file 1. **The number of reads generated for each time point in RNA isolated from caecal tissue collected from uninfected chickens and chickens 1, 2, 3, 4 and 10 days after *E. tenella* infection.

**Additional file 2. **The top 50 most significantly enriched GO categories for chicken in caecal tissue collected at 1, 2, 3, 4 and 10 days after *E. tenella* infection.

**Additional file 3. **The top 50 most significantly enriched GO categories for *E. tenella* in chicken caecal tissue collected at 1, 2, 3, 4 and 10 days after infection.

**Additional file 4. **The top 50 most significantly enriched KEGG pathways for chicken in caecal tissue collected at 1, 2, 3, 4 and 10 days after *E. tenella* infection.

**Additional file 5. **The top 50 most significantly enriched KEGG pathways for *E. tenella* in chicken caecal tissue collected at 1, 2, 3, 4 and 10 days after infection.

**Additional file 6.** Differential expression, mRNA from caeca of *E. tenella* infected vs. uninfected chickens, of A) PRR genes and B) CTL-associated genes in samples from chickens infected with 1000 *E. tenella* oocysts/bird at day 0. Point shapes indicate significance, filled triangles for FDR < 0.05 and circles for FDR > 0.05, at the indicated time point.

## Data Availability

RNA sequencing datasets generated in the current study are available in the Gene Expression Omnibus (ncbi.nlm.nih.gov/geo) under accession number GSE160169 and the Sequence Read Archive (ncbi.nlm.nih.gov/sra) under accession number SRP288913. All results on clinical signs, pathology, oocyst excretion and parasite DNA in tissues are included in this published article and raw data available from the corresponding author on reasonable request.
